# Patterns of use and appropriateness of antibiotics prescribed to patients receiving haemodialysis: an observational study

**DOI:** 10.1186/s12882-017-0575-9

**Published:** 2017-05-12

**Authors:** Katrina Hui, Michelle Nalder, Kirsty Buising, Aspasia Pefanis, Khai Y Ooi, Eugenie Pedagogos, Craig Nelson, Carl M. J. Kirkpatrick, David C. M. Kong

**Affiliations:** 1 Centre for Medicine Use and Safety, Monash University, Melbourne, Australia; 2 Department of Pharmacy, Royal Melbourne Hospital, Melbourne, Australia; 3 Victorian Infectious Diseases Service, Royal Melbourne Hospital, Melbourne, Australia; 4 The University of Melbourne, Melbourne, Australia; 5 Department of Nephrology, Western Health, Melbourne, Australia; 6 Department of Nephrology, Royal Melbourne Hospital, Melbourne, Australia; 7 Western Centre for Health Research and Education, Western Health, Melbourne, Australia

**Keywords:** Antibiotics, Dialysis, End stage renal disease, Infectious diseases, Prescribing patterns

## Abstract

**Background:**

There are limited published data on the types and appropriateness of oral and intravenous (IV) antibiotics prescribed to patients receiving haemodialysis. This information is critical to optimise antibiotic prescribing. Therefore this study aims to describe the patterns of use and the appropriateness of oral and IV antibiotics prescribed to patients receiving haemodialysis.

**Methods:**

This was a prospective, observational study across four community and two hospital inpatient haemodialysis units in Melbourne, Australia. Data were collected from July 2014 to January 2015 from participants. Antibiotic regimens prescribed were compared with nationally available antibiotic guidelines and then classified as being either appropriate, inappropriate or not assessable by an expert multidisciplinary team using the National Antimicrobial Prescribing Survey tool.

**Results:**

Overall, 114 participants consented to this study where 55.3% (63/114) received antibiotics and 235 antibiotic regimens were prescribed at a rate of 69.1 antibiotic regimens/100 patient-months. The most common oral antibiotics prescribed were amoxycillin/clavulanic acid and cephalexin. The most common IV antibiotics prescribed were vancomycin, piperacillin/tazobactam, cephazolin and ceftriaxone. The percentage of inappropriate antibiotic regimens prescribed were 34.9% (15/43) in the community setting and 22.1% (40/181) in the hospital setting. Furthermore, 29.4% (30/102) of oral and 20.5% (25/122) of IV antibiotic regimens were inappropriate with incorrect dosing as the primary reason.

**Conclusion:**

Although this study is limited by the sample size, it describes the high antibiotic exposure that patients receiving haemodialysis experience. Of concern is inappropriate dose and frequency being a major issue. This requires interventions focused on the quality use of medicines and antimicrobial stewardship aspects of prescribing in this population.

## Background

Infection contributes greatly to mortality and hospitalisation in patients receiving haemodialysis worldwide [1–3]. These patients are also at risk of developing infections caused by multi-drug resistant organisms [4, 5]. As inappropriate antibiotic use is a key driver for accelerating antimicrobial resistance there have been considerable efforts made worldwide to optimise antimicrobial use [6].

In Australia, patients receive maintenance haemodialysis in community satellite dialysis units, outpatient or inpatient hospital dialysis units or at home. Currently, many patients receive maintenance haemodialysis in community satellite or hospital outpatient units in Australia [7]. As such, these patients are likely to be managed by primary healthcare providers for minor infections, or in the hospital for more serious infections.

To date, studies have focused on investigating intravenous (IV) antibiotic prescribing and appropriateness in this population in the hospital and outpatient setting [8, 9]. However, as many of these patients are managed in the community setting, it is critical to also have good insight into appropriate oral antibiotic use. Indeed, the burden and appropriateness of antibiotics prescribed have not been well explored in this population. Such data are essential to optimise patient care and antibiotic prescribing in the haemodialysis setting, particularly in light of global concern with antimicrobial resistance. Therefore, this study aims to assess the pattern and appropriateness of oral and IV antibiotics prescribed to patients receiving haemodialysis.

## Methods

This prospective, observational study was approved by Melbourne Health (QA2014012), Western Health (QA2014.049) and Monash University (CF14/1350–2,014,000,594) ethics committees.

### Dialysis setting

Four community haemodialysis units (CHU), two community satellite and two hospital outpatient haemodialysis units, participated in this study. These CHUs were affiliated with two participating hospitals, each of which had a tertiary teaching hospital inpatient haemodialysis unit (HIHU). All CHUs and HIHUs were located in Melbourne, Australia.

### Recruitment and follow up

Eligible participants were invited to participate and written consent obtained. Eligibility criteria were: adults (≥18 years old), receiving intermittent haemodialysis and able to provide written consent. The duration of the study at each unit was four months, with a two to three week lag period between commencing the study from one participating unit to another, in order to optimise study management and participant recruitment. The overall study duration was six months (July 2014 to January 2015). All consenting participants were followed until the end of the four month study period for each unit regardless of when enrollment occurred.

In the CHU, eligible participants were approached to participate at the start of the study period by one of the investigators. For the HIHU, eligible participants were approached during their hospital stay by investigators. Any CHU participants admitted to a participating HIHU were followed up during their inpatient stay. Any HIHU participants transferred to a participating CHU were also followed up at the CHU. Participants transferred to a non-participating CHU or HIHU were not followed up.

### Data collection

Baseline demographic, medical, medication and haemodialysis-related data were collected for all participants. For each participant receiving antibiotics, the documented indication, antibiotic strength, dosage regimen, duration of treatment and specialty of the prescriber; and if available, microscopy, culture, antibiotic susceptibility (M/C/S) and therapeutic drug monitoring (TDM) results were collected. The documented indication was defined as the indication stated in the medical records, by the participant or the prescriber, irrespective of the clinical definition of the infection.

For CHU participants, data were obtained from the participant or carer and their medical histories. Participants receiving antibiotics were asked to bring their antibiotics to their haemodialysis session at the CHU. The prescriber was also contacted where possible during the study to confirm the documented indication and antibiotic regimen(s) prescribed, plus results from M/C/S tests. In the HIHU all data were obtained from the participant’s hospital medical records.

### Definitions and classifications of infections and antibiotic prescribing

The documented indications for antibiotic prescribing were classified as prophylactic therapy; respiratory tract (RTI), skin and soft tissue (SSTI), vascular access (VAI), bloodstream (BSI), urinary tract (UTI), gastrointestinal tract (GITI) and other infections; infection source not known; and infection not documented when no indication was specified [10]. Hospital-acquired infections were defined as those which occurred at least 48 h after hospital admission [11].

Only administered antibiotic regimens were evaluated in this study. Antibiotic regimens included single doses and antibiotic courses where more than a single dose was administered. A single dose is often prescribed for prophylaxis (e.g. pre-transplant prophylaxis) or empirical therapy for a suspected infection. Antibiotic regimens prescribed in the community setting were prescribed in a primary healthcare or outpatient setting. The duration of treatment in this setting was either the prescribed duration or the number of days to complete the prescribed quantity.

Antibiotic regimens prescribed in the hospital setting were those prescribed in an inpatient setting. In the hospital setting, the duration of treatment was taken as the date the antibiotic commenced to the date it was ceased on the medication chart. If the antibiotic dose, dosing interval or route of administration was altered in the medication chart, it was classified as a new antibiotic regimen.

### Assessing appropriateness of antibiotics prescribed

Oral and IV antibiotic regimens prescribed were compared to the recommendations from the Australian national Therapeutic Guidelines (TG): Antibiotic Version 14 and 15, [12, 13] unless local hospital guidelines were available, as was the case for certain antibiotics, such as vancomycin. The TG: Antibiotic is widely available in hospitals and via the majority of general practice software in Australia. A new edition of the TG: Antibiotic was released in November 2014 which included minor changes to the dosing of certain antibiotics in the haemodialysis setting. Antibiotic regimens prescribed after November 2014 were compared with the TG: Antibiotic Version 15.

The TG: Antibiotic provides recommendations from an antibiotic expert group, all of whom are working in Australia, on the types of antibiotics, dose, frequency and duration of treatment, for a wide variety of infections seen in both the community and hospital setting. The evidence to support the recommendations are drawn from published literature (where available) and the experts’ collective experiences. Antibiotic dosing guidelines in renal impairment and dialysis setting are provided as a table in an appendix in the TG: Antibiotic for all antibiotics available in Australia [13]. This table includes dose adjustments and recommendations of when an antibiotic should be dosed in relation to haemodialysis if published data is available. This guideline is available to all health professionals in either a hard copy or electronic format.

The Renal Drug Handbook Third Edition was referred to in order to confirm the ideal dosing of the antibiotic regimen when it was not available in the TG: Antibiotic or hospital guidelines [14]. Only dose adjustments for renal impairment and renal replacement therapy are provided in the Renal Drug Handbook.

Each antibiotic regimen prescribed was then reviewed by an expert multidisciplinary team to determine whether they were appropriate, inappropriate or not assessable based on the classifications utilised in the publically available National Antimicrobial Prescribing Survey (NAPS) (see Table 1) [15]. The information available for review was the documented indication, the antibiotic regimen, antibiotic allergy status and available M/C/S data. For antibiotic regimens which had M/C/S data available, if the organism(s) identified were non-susceptible to the prescribed antibiotic, these regimens were classified as inappropriate. Hospital guidelines were available for vancomycin. If vancomycin was not prescribed in accordance to the hospital guidelines or where there was no reference to TDM in the prescribed regimen, this was classified as inappropriate.

**Table 1:** Assessment of appropriateness tool based on the NAPS [15]

If evidence based guidelines are present:	Antibiotic therapy (selection, dose or frequency) concordant as per indication documented.	Optimal
	Antibiotic therapy (selection, dose or frequency), not concordant as per indication documented. However, (potential) causative pathogens will be treated/covered.	Adequate
If evidence based guidelines are absent:	Antibiotic therapy (selection, dose and frequency) will treat/cover (potential) causative pathogens as per indication documented and there is no better alternative (selection, dose and frequency) available.	Optimal
	Antibiotic therapy (selection, dose and frequency) will treat/cover (potential) causative pathogens as per indication documented, but there is a better alternative (selection, dose and frequency) available.	Adequate
Only one has to be met for all indications • Antibiotic therapy (either selection, dose and frequency) not concordant and (potential) causative pathogens will not be treated/covered • Severe hypersensitivity mismatch • Antimicrobial and/or dose and/or frequency can pose a risk of toxicity to patient • In addition for surgical prophylaxis, if duration is >24 h		Inadequate
Notes not comprehensive enough due to one or more of the following: • Patient complexity • Lack of documentation of indication • Lack of information from interventions performed, pathology, progress		Not assessable

The expert multi-disciplinary team consisted of a pharmacist with community pharmacy experience (KH), renal pharmacist (MN) and infectious diseases consultant (KB) who independently assigned appropriateness to all antibiotic regimens prescribed. The team then compared their assessments and any discrepancies were discussed until consensus was reached.

### Statistical analysis

Statistical analysis was performed using IBM SPSS Statistics version 22 and χ^2^ test, Fishers exact test and Mann-Whitney U test were used where appropriate. A *p*-value of 0.05 or less was considered statistically significant. The rate of antibiotic use is reported as the number of antibiotic regimens prescribed per 100 patient follow up months (100 PM). The patient follow up months is the sum of the months that participants were followed up.

## Results

### Patient demographics

In total, 239 patients were approached to participate in the study. Of these, 63 were excluded as they could not provide consent due to medical reasons or language barriers. Overall, 114/176 participants consented to participate with 81 from the CHU and 33 from the HIHU. Nineteen CHU participants were admitted into a participating HIHU and four HIHU participants were transferred into a participating CHU during the study period. Eight participants were considered withdrawals from the study due to death (two CHU and three HIHU participants) or kidney transplantation (one CHU and two HIHU participants). There were 340 patient-months (PM) of follow up overall, with 308 for the CHUs and 32 for the HIHUs.

The participant’s demographic data are shown in Table 2. The median (interquartile range [IQR]) age of participants was 64 (50.8–75.0) years old, 68/114 (59.7%) were males and majority were Caucasian. Diabetes was the most common reason for end-stage renal disease requiring haemodialysis. There were statistically significant differences for ethnicity, vascular access type, number of years receiving haemodialysis at enrollment and respiratory disease between CHU and HIHU participants. There were no significant differences (data not shown) between participants who were prescribed no antibiotics, appropriate antibiotic regimens and at least one inappropriate antibiotic regimen.

**Table 2:** Demographic data of study participants

Variable	Community (*n* = 81)	Hospital (*n* = 33)	Total (*n* = 114)	*p*-values
Age, median (IQR)	65 (51.5–77.5)	64 (50–74)	64 (50.8–75)	0.334
Baseline dry weight (kg), median (IQR)	71.0 (60.5–85.5)	72.0 (61.0–92.3)	72.0 (60.9–87.1)	0.413
Male (%)	46 (56.8)	20 (60.6)	68 (59.7)	0.894
Ethnicity (%)				0.039*
Caucasian	55 (67.9)	30 (90.9)	85 (74.6)	
Asian	13 (16.1)	1 (3.0)	14 (12.3)	
Other	13 (16.1)	2 (6.1)	15 (13.2)	
Primary indication of ESRD requiring HD (%)				0.922
Diabetes	34 (42.0)	12 (36.4)	46 (40.4)	
Glomerulonephritis	13 (16.1)	6 (18.2)	19 (16.7)	
Hypertension	7 (8.6)	2 (6.1)	9 (7.9)	
Polycystic kidney disease	4 (4.9)	2 (6.1)	6 (5.3)	
Other	18 (22.2)	10 (30.3)	28 (24.6)	
Unknown	5 (6.2)	1 (3.0)	6 (5.3)	
Vascular access type at enrollment (%)				0.018*
AV fistula	62 (76.5)	19 (57.6)	81 (71.1)	
AV graft	10 (12.4)	3 (9.1)	13 (11.4)	
Catheter	9 (11.1)	11 (33.3)	20 (17.5)	
Years receiving HD at enrollment, median (IQR)	3.17 (1.1–5.9)	1.5 (0.4–3.5)	2.4 (0.9–5.5)	0.005*
Current smokers (%)	11 (13.6)	4 (12.1)	15 (13.2)	1.000
Comorbidities				
Diabetes	43 (53.1)	12 (36.4)	55 (48.2)	0.105
Cardiovascular disease	78 (96.3)	30 (90.1)	108 (94.7)	0.354
Dyslipidaemia	32 (39.5)	15 (45.5)	47 (41.2)	0.558
Respiratory disease	10 (12.3)	11 (33.3)	21 (18.4)	0.009*
Previous or current diagnosis of cancer	14 (17.3)	8 (24.2)	22 (19.3)	0.393
Allergy to antibiotics (%)				1.000
Allergy to one antibiotic only:				
Pencillins	5 (6.2)	2 (6.1)	7 (6.1)	
Cephalosporins	2 (2.5)	1 (3.0)	3 (2.6)	
Other antibiotics	2 (2.5)	1 (3.0)	3 (2.6)	
Allergies to two or more antibiotics	6 (7.4)	2 (6.1)	8 (7.0)	
Previous kidney transplant (%)	11 (13.6)	9 (27.3)	20 (17.5%)	0.081
Anuric (%)	46 (56.8)	16 (48.5)	62 (54.4)	0.298

*AV* arteriovenous, *ESRD* end stage renal disease, *HD* haemodialysis, *IQR* interquartile range

*p-value <0.05

### Types of infections and microbiological culture

There were 114 documented indications for antibiotic therapy. Of the suspected and documented infections, 76 were community-acquired (rate of 22.4 episodes of infection/100 PM) and 17 hospital-acquired (5.0 episodes/100 PM). The most common infections were RTI (27/114), SSTI (19/114), BSI (14/114), UTI (11/114) and VAI (7/114). Five episodes of BSI involved participants who had catheters as their vascular access. Additionally, 16 indications were for prophylactic therapy of which, five were for surgical prophylaxis and five for *Pneumocystis jiroveci* pneumonia prophylaxis. Infection was not documented for three episodes and two had unknown indications due to missing medical inpatient records.

There were 190 microbiological cultures requested with blood (97/190), wound (28/190) and urine cultures (24/190) the most common. Most were requested from the hospital setting, with three from the community setting. Seventy micro-organisms were isolated from 54 cultures; 35/70 were gram-positive and 17/70 were gram-negative bacteria. The most common gram-positive bacteria isolated were *Staphylococcus* (23/35) and *Enterococcus* (7/35) species. Three were methicillin-resistant *Staphylococcus aureus* (MRSA) and one was vancomycin-resistant enterococcus. The most common gram-negative bacteria isolated was *Escherichia coli* (4/17). No multi-drug resistant gram-negative bacteria were isolated.

### Antibiotic use

Overall, 55.3% (63/114) of participants received antibiotics; 43.2% (35/81) CHU participants and 84.8% (28/33) HIHU participants. A total of 235 antibiotic regimens were prescribed (110 oral and 125 IV). The rate of antibiotic use overall was 69.1 antibiotic regimens/100 PM, with 32.4 regimens/100 PM for oral and 36.8 regimens/100 PM for IV antibiotics. The rate of antibiotic regimens prescribed in the community setting was 15.5 regimens/100 PM (48 antibiotic regimens in 308 PM) and in the hospital setting the rate was 584.4 regimens/100 PM (187 antibiotic regimens in 32 PM). Only four regimens in the community setting and 29 in the hospital setting were prescribed as directed therapy after a pathogen had been reported in clinical specimens. Twenty-one antibiotic regimens were prescribed for prophylactic therapy, eight of which were trimethoprim/sulfamethoxazole.

The most common oral antibiotics prescribed were amoxycillin/clavulanic acid for RTI and SSTIs and cephalexin for SSTI and UTIs (Table 3). The most commonly prescribed IV antibiotics were vancomycin, piperacillin/tazobactam, cephazolin and ceftriaxone (Table 3). Vancomycin and cephazolin were commonly prescribed for BSI, SSTI and VAIs; piperacillin/tazobactam for SSTIs; and ceftriaxone for RTIs.

**Table 3:** The 15 most commonly prescribed antibiotic regimens

	Antibiotic	Indications	Setting prescribed		Route of administration		Total	No. (%) inappropriate	Reasons for inappropriate classification
			Community	Hospital	IV	Oral			
1	Vancomycin	BSI, SSTI, VAI	2	30	32	-	32	3 (9.4)	Incorrect dose/frequency
2	Piperacillin/tazobactam	SSTI	-	23	23	-	23	1 (4.3)	Surgical prophylaxis >24 h
3	Amoxycillin/clavulanic acid	RTI, SSTI	5	15	-	20	20	7 (35.0)	Incorrect dose/frequency, spectrum too broad, incorrect duration
4	Cephazolin	BSI, SSTI, VAI	-	18	18	-	18	10 (55.6)	Incorrect dose/frequency, allergy mismatch, spectrum too narrow, unnecessary antibiotic therapy
5	Ceftriaxone	RTI	-	16	16	-	16	2 (12.5)	Incorrect dose/frequency, spectrum too narrow
6	Amoxycillin	RTI, BSI	11	5	4	12	16	3 (18.8)	Incorrect dose/frequency, spectrum too narrow, incorrect duration
7	Cephalexin	SSTI, UTI	12	2	-	14	14	6 (42.9)	Incorrect dose/frequency, spectrum too broad, microbiology mismatch
8	Trimethoprim/sulfamethoxazole	PR	2	11	-	13	13	4 (30.8)	Incorrect dose/frequency
9	Doxycycline	RTI	1	12	-	13	13	0 (0.0)	-
10	Ciprofloxacin	SSTI, RTI	-	11	1	10	11	4 (36.4)	Incorrect dose/frequency, microbiology mismatch, spectrum too narrow
11	Flucloxacillin	VAI, SSTI	1	8	5	4	9	2 (22.2)	Incorrect dose/frequency
12	Meropenem	RTI, BSI	-	7	7	-	7	4 (57.1)	Incorrect dose/frequency
13	Metronidazole	SSTI	1	5	3	3	6	2 (33.3)	Incorrect dose/frequency, unnecessary antibiotic
14	Azithromycin	RTI	-	5	5	-	5	0 (0.0)	-
15	Trimethoprim	UTI	2	2	-	2	4	4 (100.0)	Incorrect dose/frequency, incorrect duration

*BSI* blood stream infection, *IV* intravenous, *PR* prophylactic therapy, *RTI* respiratory tract infection, *SSTI* skin and soft tissue infection, *UTI* urinary tract infection, *VAI* vascular access infection

### Appropriateness of antibiotics prescribed

Overall, 95.3% (224/235) of the antibiotic regimens could be assessed for appropriateness of prescribing. Of these, 75.4% (169/224) were appropriate and 24.6% (55/224) were inappropriate (Table 4). The rate of inappropriately prescribed antibiotic regimens was 16.2 regimens/100 PM. Of the appropriate regimens, 93 were compliant with TG: Antibiotics, 42 with local guidelines, one with the Renal Drug Handbook. Guidelines were not available for 33 antibiotic regimens, but were deemed appropriate by all assessors using the classification provided in Table 1. In the community setting, 34.9% (15/43) of the antibiotics regimens were classified as inappropriate compared to 22.1% (40/181) in the hospital setting. Additionally, 29.4% (30/102) of oral and 20.5% (25/122) of IV antibiotic regimens were classified as inappropriate. Antibiotic regimens were commonly classified as inappropriate due to incorrect dose or frequency selected (Table 4). For the majority, the dose of the antibiotic was either too high or the dosing interval too short.

**Table 4:** The appropriateness of the antibiotic regimens prescribed

Classification	Setting		Route of administration		Total
	Community	Hospital	Oral	IV	
Total assessable	43/48 (89.6)	181/187 (96.8)	102/100 (92.7)	122/125 (97.6)	224/235 (95.3)
Optimal	13/43 (30.2)	97/181 (53.6)	42/102 (41.2)	68/122 (55.7)	110/224 (49.1)
Adequate	15/43 (34.9)	44/181 (24.3)	30/102 (29.4)	29/122 (23.8)	59/224 (26.3)
Inadequate	15/43 (34.9)	40/181 (22.1)	30/102 (29.4)	25/122 (20.5)	55/224 (24.6)
Reasons for inadequate classification					
Incorrect dose or frequency	14/15 (93.3)	29/40 (72.5)	26/30 (86.7)	17/25 (68.0)	43/55 (78.2)
*Dose too high*	5/14 (35.7)	7/29 (24.1)	8/26 (30.8)	4/17 (23.5)	12/43 (27.9)
*Dose too low*	1/14 (7.1)	1/29 (3.4)	1/26 (3.8)	1/17 (5.9)	2/43 (4.7)
*Dosing interval too short*	5/14 (35.7)	17/29 (58.6)	13/26 (50.0)	9/17 (52.9)	22/43 (51.2)
*Dosing interval too long*	3/14 (21.4)	4/29 (13.8)	4/26 (15.4)	3/17 (17.6)	7/43 (16.3)
Incorrect duration	3/15 (20.0)	2/40 (5.0)	5/30 (16.7)	-	5/55 (9.1)
Allergy mismatch	-	4/40 (10.0)	-	4/25 (16.0)	4/55 (7.3)
Spectrum too narrow	1/15 (6.7)	3/40 (7.5)	2/30 (6.7)	2/25 (8.0)	4/55 (7.3)
Spectrum too broad	3/15 (20.0)	-	3/30 (10.0)	-	3/55 (5.5)
Unnecessary antibiotic therapy	-	3/40 (7.5)	1/30 (3.3)	2/25 (8.0)	3/55 (5.5)
Microbiology mismatch	-	2/40 (5.0)	2/30 (6.7)	-	2/55 (3.6)
Surgical prophylaxis >24 h	-	1/40 (2.5)	-	1/25 (4.0)	1/55 (1.8)

All data shown as number of antibiotic regimens and percentage. Reasons for inadequate classification are not mutually exclusive

IV - intravenous

The most common inappropriately prescribed oral antibiotics were amoxycillin/clavulanic acid and cephalexin (Table 3). For amoxycillin/clavulanic acid, often the dose prescribed was too high or dosing interval too long. In comparison, the main reason for cephalexin regimens being classified as inappropriate was due to dosing interval being too short. Additionally, all trimethoprim regimens were classified as inappropriate as the dose (300 mg) prescribed was too high given guidelines recommend 150 mg daily [13].

Both cephazolin and meropenem were the most common inappropriately prescribed IV antibiotics (Table 3) often due to the dosing interval being too short. Almost all the vancomycin regimens prescribed were classified as appropriate, with only three regimens classified as inappropriate due to dosing frequency with no reference to TDM. Of the three inappropriately prescribed vancomycin regimens, TDM was not available for two of the regimens.

## Discussion

To the best of our knowledge, this study is the first to evaluate the types and appropriateness of antibiotic regimens prescribed to patients receiving haemodialysis in Australia, and more specifically, for oral antibiotics, worldwide. In this study, 55% of the participants received at least one antibiotic in comparison to the Australian general population where 45% were supplied with at least one antibiotic in a twelve month period [16]. The rate of antibiotic use was 69.1 antibiotic regimens/100 PM suggesting a high burden of antibiotic exposure in this cohort. Other studies have reported an antimicrobial use rate of 39.3 doses/100 PM [8] and 12.0 courses/1000 dialysis days (5111 antibiotic courses in 424,700 dialysis days) [9]. As patients receiving haemodialysis are at risk of developing infections and antibiotic exposure is high, it is imperative that antibiotics are used and prescribed optimally in these patients.

Classifying an antibiotic as inappropriate was often related to the dose and dosing interval selected. This has also been reported in patients with chronic kidney disease not requiring dialysis [17]. Consequently, adverse effects, toxicity and waste of healthcare resources could occur with higher or more frequent dosing, or treatment failure and/or an increased risk of antibiotic resistant bacteria with suboptimal dosing. Also of concern were the 15 regimens where the antibiotic selected was inappropriate due to the spectrum being too narrow or broad, microbiology or allergy mismatch or the antibiotic was unnecessary (Table 4) based on the documented indication, microbiology and sensitivity results, allergy status of the patient and taking into account the other antibiotics prescribed. Inappropriate selection of antibiotics is concerning as it can contribute to antibiotic resistance. In the case where the spectrum is too narrow or there is an allergy mismatch, it has the potential to impact adversely on patient outcomes.

To date, very few studies have explored antibiotic use and appropriateness in patients receiving haemodialysis. A hospital outpatient based study from the United States of America evaluated the appropriateness of IV antibiotics prescribed [8]. They found that one third of antibiotic doses were inappropriate due to either: criteria for infection not met, a narrower spectrum antibiotic not chosen or indication for surgical prophylaxis not met [8]. Although our study design was different to Snyder et al. and thus cannot be compared directly, our study observed that a quarter of antibiotic regimens prescribed were classified as inappropriate.

Oral antibiotics are commonly prescribed in the primary care and hospital setting, yet are less well explored compared to IV antibiotics. Of concern is that almost a third of oral antibiotic regimens prescribed in this study were inappropriate, highlighting the need for more research to further explore the use and appropriateness of oral antibiotics prescribed to this patient population in other countries.

Interestingly, vancomycin was prescribed appropriately in most cases in our study. Vancomycin was prescribed empirically for most cases in the study, which was deemed an appropriate indication due to the risk of MRSA infections in this patient group. Other studies where vancomycin is prescribed empirically before culture and sensitivities are available have also reported that 80–88% of vancomycin doses or courses are appropriate [18, 19]. Yet, once culture and sensitivities are available, vancomycin is commonly classified as inappropriate due to not fitting the criteria for infection or for failing to prescribe a narrower spectrum antibiotic [8, 18, 19]. This was not observed in the current study which may be due to the small numbers of vancomycin regimens prescribed.

There are unique challenges associated with prescribing antibiotics in the haemodialysis setting such as the need to take into account the effect of haemodialysis on antibiotic clearance. It is vital that prescribers have readily accessible user-friendly resources to aid antibiotic prescribing to those receiving haemodialysis. Examples include decision support programs or incorporation of guidelines or tools into clinical and health pathways. Dissemination of knowledge from specialists in this area to other prescribers, particularly in primary healthcare could also be explored.

Establishing antimicrobial stewardship in dialysis centres has been discussed by D’Agata [20]. The majority of published data regarding antimicrobial stewardship programs and its implementation in general, have occurred in a hospital setting. In Australia and other parts of the world, a large proportion of patients receiving haemodialysis are managed in the community. It may be quite challenging to develop an antimicrobial stewardship program targeted at dialysis units located in the community as anecdotally, these patients will often not notify the dialysis unit staff that they are taking antibiotics, nor are the dialysis staff prescribing or often administering antibiotics, particularly oral antibiotics, to patients at the dialysis units. However, it may be worthwhile to provide further continuing education to general practitioners, community pharmacists or nurses on antibiotic prescribing and use in this particular population.

Whilst the study was conducted in Australia, it is acknowledged that there may be differences with regards to guideline recommendations for antibiotic dosing in the dialysis setting in other countries. In Australia, the main national antibiotic guideline is the TG: Antibiotics which provides recommendations for dose adjustments to be made in renal impairment. Other international guidelines available to guide the dosing of antibiotics in the dialysis setting include the Renal Drug Handbook, published in the United Kingdom, [14] and the Sanford Guide to Antimicrobial Therapy published in the United States [21]. Although these references provide recommendations on how certain antibiotics should be dosed in the dialysis population, there are a number of differences with regards to the recommendations made for certain antibiotics. For example, the dosage recommendations for amoxycillin/clavulanic acid for patients receiving haemodialysis as per the three aforementioned guidelines are 500/125 mg every 12 h (TG: Antibiotics), [13] 250/125 mg or 500/125 mg three times daily (Renal Drug Handbook) [14] and 250/125 mg or 500/125 mg once daily with an extra dose after dialysis (Sanford Guide to Antimicrobial Therapy) [21]. Such differences could be due to a number of factors such as: the types of patients encountered, differences in microbiological antibiotic susceptibility profiles, different interpretations of the product information recommendations, a lack of published data to inform the dosing of certain antibiotics in the dialysis setting, clinical experience and availability of antibiotics and antibiotic strengths in each country. In addition, many hospitals will also have their own “in-house” guidelines and protocols on how antibiotics are to be dosed in the dialysis setting and dosing practices may also vary between hospitals.

## Limitations

Despite sample size limitations and a shorter follow up period compared to previous studies, [8, 9] the current study provides useful information related to antibiotic prescribing to patients receiving haemodialysis, particularly in the Australian setting. Our findings are likely to be conservative as many patients who either elected not to take part, or were excluded, were also likely to be prescribed antibiotics during the study period. Also, whilst the findings may not be generalisable to other countries as all six dialysis units were located in Australia, the results do highlight the need for more research to be done in the haemodialysis population elsewhere. This study did not capture reported adverse effects, adherence to the prescribed antibiotic regimens, reasons or causes for inappropriate antibiotic prescribing and patient specific outcomes. Lastly, the impact of the revised TG: Antibiotics (Version 15) on the results of this study is likely to be negligible as there were minor changes to very few antibiotic regimens in the haemodialysis setting between the two versions.

## Conclusion

The current study has provided important insight into the types and appropriateness of antibiotics prescribed to patients receiving haemodialysis in an environment where data are scant. Indeed, for the first time, the appropriateness of orally administered antibiotics prescribed to this cohort was investigated. Our data indicates that there is a high burden of antibiotic exposure and considerable inappropriate prescribing of antibiotics to those receiving haemodialysis. Our study suggests that antimicrobial stewardship or continuing education related interventions are needed to optimise the use of antibiotics in patients receiving haemodialysis to minimise the emergence of resistant organisms and importantly, to optimise patient safety and outcomes.

## Abbreviations

BSI: Bloodstream infection
CHU: Community haemodialysis unit
GITI: Gastrointestinal tract infection
HIHU: Hospital inpatient haemodialysis unit
IV: intravenous
M/C/S: Microscopy, culture and sensitivities
MRSA: Methicillin-resistant *Staphylococcus aureus*
NAPS: National Antimicrobial Prescribing Survey
PM: Patient month
RTI: Respiratory tract infection
SSTI: Skin and soft tissue infection
TDM: Therapeutic drug monitoring
TG: Therapeutic guidelines
UTI: Urinary tract infection
VAI: Vascular access infection
